# Current treatment strategies for patients with advanced gastroenteropancreatic neuroendocrine tumors (GEP-NETs)

**DOI:** 10.1186/s40842-018-0066-3

**Published:** 2018-07-11

**Authors:** Inbal Uri, Simona Grozinsky-Glasberg

**Affiliations:** Neuroendocrine Tumor Unit, Endocrinology and Metabolism Department, Division of Medicine, Hadassah-Hebrew University Medical Center, P.O.B. 12000, 91120 Jerusalem, Israel

**Keywords:** Treatment, Neuroendocrine tumor

## Abstract

**Background:**

Neuroendocrine tumors (NETs) are rare neoplasms, with an estimated annual incidence of ~ 6.9/100,000. NETs arise throughout the body from cells of the diffuse endocrine system. More than half originate from endocrine cells of the gastrointestinal tract and the pancreas, thus being referred to as gastroenteropancreatic NETs (GEP NETs). The only treatment that offers a cure is surgery, however most patients are diagnosed with metastatic disease, and curative surgery is usually not an option.

Since the majority of patients are not candidate for curative surgery, they can be offered long-term systemic treatment, for both symptomatic relief and tumor growth suppression. Evidence based treatment options include somatostatin analogues, everolimus (an mTOR inhibitor), sunitinib (a tyrosine kinase inhibitor), peptide receptor radionuclide therapy (PRRT), chemotherapy, etc., alone or combined with cytoreductive procedures (surgery or liver directed procedures). However, there is an increasing need for novel therapies. Other treatment options being investigated are immunotherapy and epigenetic assessment that may lead to more personalized interventions. Following first line therapy with somatostatin analogues, there is no clear information to date indicating a preferred treatment sequence, and therefore the treatment approach should be individualized based on each NET patient characteristics.

**Conclusions:**

NET patients are increasingly diagnosed throughout the world, usually with metastatic disease and requiring systemic therapy. We believe that each patient should be therefore thoroughly evaluated and individually discussed by a multidisciplinary and dedicated NET-expert team, updated with all treatment options including ongoing clinical trials, and before selecting the proper treatment sequence.

## Background

Neuroendocrine tumors (NETs) are rare neoplasms, with an estimated annual incidence of 6.9/100,000, arising from cells of the diffuse endocrine system [1], mainly dispersed throughout the digestive system and respiratory tract. More than half arise inside the gastrointestinal tract and the pancreas and are referred to as gastroenteropancreatic NETs (GEP NETs) [2].

Most NETs grow slowly over the years, and their symptoms are related to tumor mass (non-functioning, NF-PNETs); however, in about 30%, symptoms related to the hypersecretion of hormones from the tumor may occur (functioning, F- PNETs). F-PNETs may secret insulin, glucagon, gastrin, vasoactive intestinal polypeptide, etc., with clinical pictures as by hormonal hypersecretion (hypoglycemia, hyperglycemia, peptic ulcers, secretory diarrhea, etc.), and may require systemic treatment for both tumor and symptom control, when not resectable [3]. Histo-pathologically, most GEP NETs are well-differentiated tumors and are divided into grade 1 (G1, with a Ki67 proliferation index of ≤3%), and grade 2 (G2, with a Ki67 proliferation index between 3 and 20%). A smaller percentage are represented by grade 3 (G3) tumors with a Ki67 greater than 20% [4]. However, it is believed that within the G3 group, the biological behavior of tumors with a Ki67 index between 20 and 55% is less aggressive than tumors with a Ki67 index above 55% [3].

**Fig. 1:**
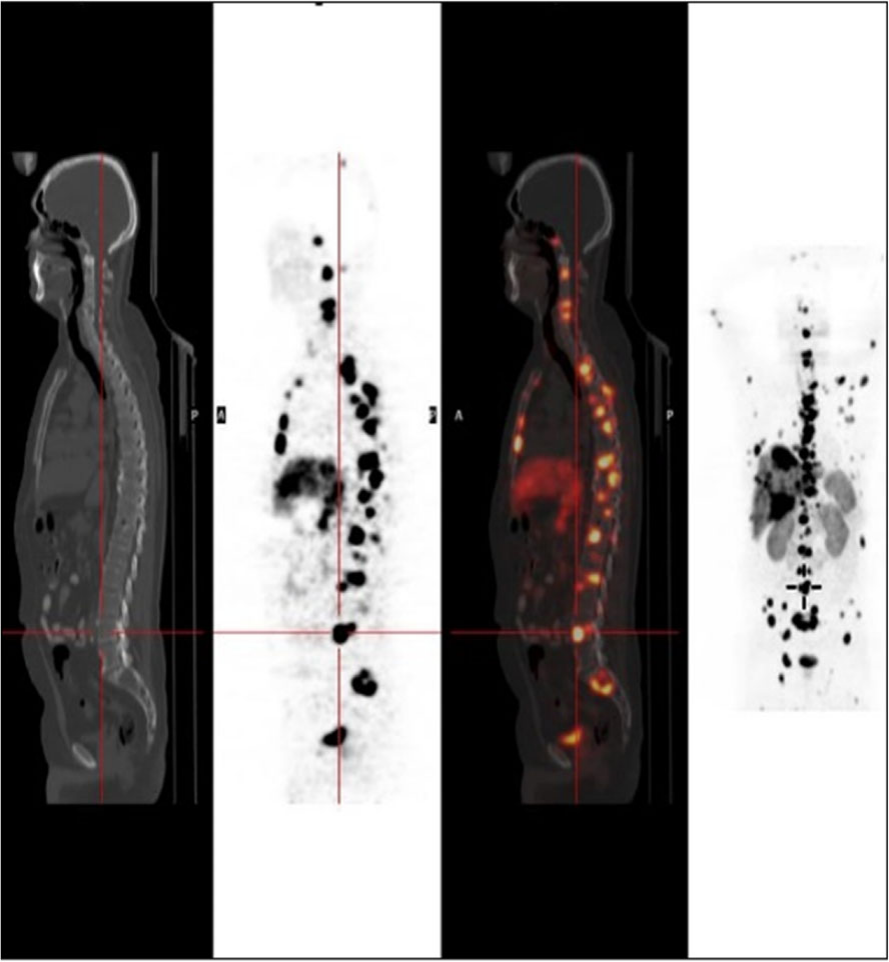
Ga68-DOTATATE-PET/CT images of a patient with metastatic G1 intestinal NET to the liver, bones and lymph nodes – showing high uptake be the tumor, in correlation with increased expression of somatostatin receptors, mainly SSTR2

Most neuroendocrine tumors typically express high levels of somatostatin receptors, therefore making the somatostatin receptor imaging a useful tool for the diagnosis and staging of the disease in these patients (Fig. 1). It is recommended to use Ga-68 DOTATATE PET/CT imaging whenever available, as it shows a higher sensitivity and specificity for detecting NETs compared with In-111 DTPA-octreotide, and other conventional imaging [5].

Because of their marked heterogeneity, the treatment of NETs is challenging, and a multidisciplinary approach is mandatory for maximizing patient prognosis and survival.

The only curative treatment for NETs is surgery. However, most NETs are diagnosed when the disease is advanced and metastatic, and as such are not amenable to curative surgery, which leads to the need for systemic therapies.

In the present manuscript, we thoroughly summarize the treatment options for patients with advanced G1&G2 GEP-NETs, to date.

### Biological therapies

#### Somatostatin Analogues (SSAs)

Somatostatin (SST) is a neuropeptide secreted from paracrine cells along the gastrointestinal tract and in the brain. It exerts its effects by binding to five receptors coupled to G-protein (SST receptor 1 to 5, SSTR1–5) [6]. It inhibits secretion of many hormones, acts as an immune regulator, as a neurotransmitter [7] and also has cytotoxic and cytostatic actions and may induce apoptosis under special conditions [2].

The SSA antiproliferative effect is mediated through several mechanisms, either as a direct effect on tumor cells by binding to SSTR (inducing cell cycle arrest, a pro-apoptotic effect), or indirect effects through inhibition of angiogenesis, hormones secretion, and immunomodulation [8]. Most GEP-NETs overexpress somatostatin receptors, mainly SSTR2 [7].

The somatostatin analogue (SSA) octreotide has been in use since the 1980s, in its short acting formulation, administered subcutaneously at a dose of 150 mcg 3 times daily, and showed clinical improvement in up to 88% of patients [9]. Later on, octreotide LAR (long acting release) was introduced, as a once a month intramuscular injection, making the treatment more convenient.

Initially, the antiproliferative effect of SSAs was demonstrated in several retrospective studies. The PROMID study was the first prospective placebo-controlled, double-blind, phase III study that demonstrated, in an unequivocal manner, statistically significant prolongation of time to tumor progression in patients with well differentiated metastatic intestinal NETs, treated with octreotide LAR, compared with placebo (14.3 vs 6 months). Importantly, both functioning (defined as the presence of carcinoid syndrome and increased urinary 5-hydroxyindole acetic acid) and nonfunctioning tumors, responded similarly, with a trend for a better effect in patients with lower hepatic tumor burden [10].

The CLARINET study, another prospective randomized, double-blind, placebo-controlled study, showed similar findings and confirmed the PROMID results however this time in a larger cohort of patients with both intestinal and pancreatic nonfunctioning and progressive NETs. The study demonstrated a statistically significant prolongation of progression free survival (PFS) (median not reached vs. median of 18.0 months), with another long acting SSA, lanreotide Autogel, compared with placebo [11].

Higher then labeled dosages of SSAs may be used in selected patients when symptoms control is not achieved, and also for better tumor control in slow growing tumors, and before proceeding to other systemic treatments [12].

Pasireotide (SOM230) is a novel multireceptor SSA, showing high affinity to SSTR 1,2,3&5. It is approved for treating acromegaly resistant to octreotide and lanreotide, and was also investigated for treatment of NETs [9]. A recent phase III trial comparing pasireotide LAR and octreotide LAR in patients with carcinoid syndrome not controlled on SSA, showed no advantage of pasireotide regarding symptom control, but an improvement in PFS (11.8 vs 6.8 months) was noted, although it was not statistically significant [13].

SSAs are usually well tolerated and with limited side effects, the more frequent being pain in the injection site and gastrointestinal side effects (abdominal pain, diarrhea, nausea) [7].

The development of somatostatin analogues (SSA) as an important treatment tool has revolutionized the clinical management of patients with NETs. However, although symptomatic relief and stabilization of tumor growth for various periods are observed in many patients treated with SSA, tumor regression is rare, and therefore combined therapeutic strategies are needed to further improve the clinical management of patients with advanced NETs.

#### Interferon (IFN) alpha

Interferon (IFN) alpha was introduced in the treatment of NETs in the 1980s by Öberg and colleagues [14]. It has several mechanisms of action on cell proliferation and differentiation [8].

Over 30 studies including hundreds of NETs patients treated with IFN, with treatment periods of 39 ± 35 weeks in median, showed symptoms relief in up to 70% of the cases and biochemical response in up to 60%, with tumor stabilization for limited periods in most of the patients. However, the treatment with IFN is limited by challenging side effects: from flu-like symptoms, lasting for several days after treatment, to chronic fatigue, liver toxicity, bone marrow suppression, depression, and autoimmune-related conditions, mainly thyroiditis [15]. In general, the treatment with IFN is reserved for patients who are resistant to, or cannot tolerate, SSA and other systemic therapies, in addition to SSA for better symptom control, or as a bridge before other treatments are initiated [12].

#### Telotristat ethyl

Telotristat ethyl is a novel oral inhibitor of tryptophan hydroxylase, the rate-limiting enzyme in serotonin biosynthesis. Two early-stage clinical studies of telotristat ethyl demonstrated evidence of clinical activity in carcinoid syndrome and a favorable safety profile, with minimal CNS activity [16, 17].

A recent prospective randomized phase III study demonstrated statistically significant reduction in the frequency of bowel movements in parallel with reductions in the main serotonin metabolite 5-hydroxyindoleacetic acid (5HIAA) in the urine of patients with uncontrolled carcinoid syndrome treated with telotristat, in a dose dependent manner [18]. Telotristat appears to have a favorable side effects profile, with mild nausea, abdominal discomfort, and mild elevation of liver transaminases most frequently reported. Based on these promising results, the U.S. Food and Drug Administration (FDA) recently approved the use of telotristat ethyl (Xermelo, Lexicon Pharmaceuticals, Inc.) as the first and only oral treatment, in combination with SSAs, for adult patients with carcinoid syndrome-related diarrhea inadequately controlled by SSA therapy alone.

### Targeted therapies

#### Mammalian (mechanistic) Target of Rapamycin (mTOR) inhibitors

mTOR is a serine/threonine protein kinase, that regulates several cellular processes such as cell growth, proliferation and survival [19]. Abnormal over-activation of mTOR was observed in many cancer models including NETs, and inhibition of mTOR by rapamycin and its analogues such as RAD001, known as everolimus (Afinitor, Novartis Oncology), was shown to arrest tumor cell proliferation and to slow the tumor growth. Interestingly, preclinical studies demonstrated that susceptibility to everolimus may vary in individual patients even if the tumor has the same site of origin [19, 20].

Following these observations, the RAD001 in Advanced Neuroendocrine Tumors (RADIANT) trial program has been developed (Table 1). Three phase III prospective, randomized, double-blind placebo-controlled trials in G1 & G2 NET patients with progressive disease receiving depot octreotide were developed, based on the encouraging findings of the phase II RADIANT-1 [21]: RADIANT-2, in patients with advanced mainly intestinal NETs; RADIANT-3, in patients with advanced pancreatic NETs; and RADIANT-4, in patient with progressive NETs originating in the lung or gastrointestinal tract [22–24].

**Table 1:** A summary of currently used therapy modalities (systemic and liver loco-regional) for advanced GEP NETs, indicating reference studies for each one, and frequent side effects

Drug	Study	Population (n)	Design	Primary end-point	Main Outcome	Major side effects
Octreotide LAR	Rinke et al. 2009 [10], PROMID study	Intestinal NETs F/NF (85)	Randomized phase III	PFS	14.3 months vs 6 months with placebo	Diarrhea, flatulence, cholelithiasis
Lanreotide autogel	Caplin et al. 2014 [11], CLARINET study	NF Intestinal/ Pancreatic NETs (204)	Randomized phase III	PFS	Median not reached vs 18 months with placebo	Diarrhea, flatulence, cholelithiasis, hyperglycemia
Interferon	Oberg et al. 2012 [15]	GEP NETs, carcinoid syndrome	Review	Clinical and biochemical response, tumor effect	Symptoms relief up to 70%, biochemical response 50–60%, SD up to 70%	Flu-like symptoms, chronic fatigue, liver toxicity, bone marrow suppression, depression, autoimmune-related conditions
Everolimus	Yao et al., 2008 [21], RADIANT-1 study	PNET (160)	Single-arm (± sandostatin) phase II	Response rate	8.7% objective response rate; 84.7% stable disease	
	Pavel et al., 2011 [23], RADIANT-2 study	Intestinal NETs (420)	Randomized phase III	PFS	16.4 months vs 11.3 months with placebo	Stomatitis, rash, fatigue, diarrhea, nausea, infections, fever, cytopenia, edema, hyperglycemia, dyspnea, pneumonitis
	Yao et al., 2011 [23], RADIANT-3 study	PNETs (410)	Randomized phase III	PFS	11 months vs 4.6 months with placebo	
	Yao et al. 2016 [24], RADIANT-4 study	Lung/Intestinal NETs (302)	Randomized phase III	PFS	11 months vs 3.9 months with placebo	
Sunitinib	Raymond et al. 2011	Pancreatic NETs (171)	Randomized phase III	PFS	11.4 months vs 5.5 months with placebo	Diarrhea, nausea, asthenia, vomiting, fatigue, HTN, neutropenia, stomatitis, palmar-plantar erythrodysesthesia
Telotristat ethyl	Pavel et al. 2015 [16], TELESTAR study	Carcinoid syndrome (135)	Randomized phase III	Reduction in daily bowel movements	Mean reduction of 1.7–2.1 BM/day (dose dependent) vs 0.9 with placebo	Nausea, abdominal pain, vomiting, fatigue, infections, increased LFTs
PRRT	Strosberg et al. 2017 [33], NETTER-1 trial	Intestinal NETs (229)	Randomized phase III	PFS	PFS at 20 months 65.2% vs 10.8% with SSA alone	Nausea, vomiting, renal impairment, marrow toxicity
STZ-5FU	Dilz et al. 2015 [40]; Clewemar et al. 2016 [41]	pNETs (96) pNETs (133)	Retrospective	PFS	19.4 months 23 months	Nausea, fatigue, kidney toxicity, bone marrow suppression
CAPTEM	Strosberg et al. 2011 [45]; Fine et al. 2013 [46]	pNETs (30) GEP NETs (18)	Retrospective	PFS	18 months 14 months	Fatigue, nausea, myelosuppression, palmar-plantar erythrodysesthesia
TACE	Grozinsky-Glasberg et al. 2018 [54]	NETs (57)	Retrospective	PFS	14 months	Fever, leukocytosis, abdominal pain, nausea, elevated liver enzymes (post embolization syndrome), carcinoid crisis, liver failure, cholecystitis, liver abscess
SIRT	Kennedy et al. [55]	NETs (158)	Retrospective	Imaging response	SD 22.7%, PR 60.5%, CR 2.7%, PD 4.9%	Fatigue, nausea, pain, ascites,

*F* functioning, *NF* non-functioning, *PFS* progression free survival, *PRRT* peptide receptor radionuclide therapy, *STZ-5FU* streptozotocin + 5-fluorouracil, *CAPTEM* capecitabin + temozoomide, *TACE* trans-arterial chemoembolization, *SIRT* selective interval radiation therapy, *HTN* hypertension, *LFT* liver function tests

Based on significant prolongation of median progression-free survival (PFS) with everolimus versus placebo (11 months vs 4.6 months respectively; RADIANT-3), the drug was initially approved by FDA in PNETs. More recently, everolimus was approved also in non-functional progressive intestinal and lung NETs, based on similar results shown by the RADIANT-4 study (median PFS 11 months with everolimus vs 3.9 months with placebo). Importantly, the combination of everolimus and SSAs is believed to have a synergistic effect, and therefore these drugs are usually used in combination in patients with progressive NETs.

The safety profile of everolimus is challenging, with around 60% of patients requiring dose reduction and up to 19% requiring therapy withdrawal due to side effects, such as stomatitis, rash, diarrhea, fatigue, weight loss, hyperglycemia, upper respiratory tract infections, etc. [22, 24].

Noteworthy, clinical trials exploring everolimus in G3 NETs are ongoing (EVINEC- NCT02113800, NCT02248012; www.clinicaltrials.gov ). Moreover, everolimus significantly prolonged median PFS, regardless of prior chemotherapy, in PNETs patients [25].

#### Tyrosine Kinase Inhibitors (TKI)

Abnormal regulation of angiogenesis was found to be an important process in the growth and metastatic spread of GEP-NETs, strongly related to overexpression of vascular endothelial growth factor (VEGF), and of other growth factors and their tyrosine kinase receptors. Drugs that inhibit these receptors and pathways are therefore the new therapeutic directions for patients with advanced GEP-NETs [26].

Sunitinib maleate (Sutent®, Pfizer, Inc.) is a tyrosine kinase inhibitor (TKI), that can irreversibly inhibit several kinases including the VEGFR family, with anti-tumoral and antiangiogenic effect against several solid tumors (e.g., renal cell carcinoma and gastrointestinal stromal tumors). Sunitinib was proven effective for pNETs in both preclinical and clinical studies [27].

In a multinational, randomized, double-blind, placebo controlled phase III trial, 171 patients with well differentiated PNETs, who had evidence of disease progression, received sunitinib or placebo and best supportive care [28]. The safety monitoring committee recommended early discontinuation of the trial, after recognizing more deaths and serious adverse events, and shorter PFS in the placebo group, whereas there was a statistically significant prolongation of PFS (11.4 vs 5.5 months) and of the overall survival (9 vs 21 deaths), in the sunitinib group. The adverse events profile of sunitinib is complex, and includes diarrhea, nausea, vomiting, and fatigue; less frequent are hypertension, palmar-plantar erythrodysesthesia, neutropenia, hypothyroidism etc. Based on this study, the drug was approved for the treatment of locally advanced or metastatic PNETs. There is still no clear evidence of efficacy of tyrosine kinase inhibitors in non-pancreatic NETs.

Pazopanib and axitinib are multi-targeted kinase inhibitors of VEGF receptors 1–3. In phase II studies, pazopanib has shown some effect in patients with PNETs, and axitinib in patients with advanced progressive extra-pancreatic NETs [29, 30].

### Peptide receptor radionuclide therapy (PRRT)

Most GEP-NETs express somatostatin receptors (predominantly SSTR2 & SSTR5), permitting both tumor visualization and treatment with radiolabeled somatostatin analogues [31] (Fig. 1).

Peptide receptor radionuclide therapy (PRRT), either with 90-Yttrium-labeled compounds or more recently with 177-Lu-DOTATATE, have been used for the past 15 years in uncontrolled trials in a variety of NET patients. They have demonstrated disease stabilization in most, and tumor remissions in up to 15–35% of patients, in different studies [32].

PRRT with 177-Lu-DOTATATE is currently the most widely used. Recently, the phase III NETTER-1 trial evaluated the efficacy and safety of 177-Lu-DOTATATE (compared with high dose octreotide LAR), in patients with advanced SSTR positive intestinal NETs, who progressed on octreotide LAR. The study demonstrated a significant tumor response rate of 18% in the PRRT group compared with 3% in the control, together with 79% risk reduction for disease progression or death [33].

Prognostic factors for better response to PRRT are higher SSTR expression, defined by higher uptake on imaging: grade 3–4 uptake in octreoscan by Krenning score, and maximal SUV of > 16 by 68Ga-DOTATOC PET CT. Other parameters are tumor origin (pNETs apparently being the tumors that respond most), lower tumor burden (both primary tumor and hepatic spread), and patient performance status [34, 35].

Usually well-tolerated, acute and subacute side effects from PRRT may include nausea and/or vomiting (attributed usually to the co-administration of amino acids for renal protection, and occurring during or shortly after treatment), carcinoid crisis (rare, in less than 1% of the patients), bone marrow suppression, occurring 4–6 weeks after treatment, and renal function deterioration. The most acute and subacute side effects are self-limiting. Theoretically, there can be hepatic toxicity, especially in high burden of liver metastases [35]. Several large cohorts have evaluated the long-term tolerability of PRRT. Severe renal failure is rare (reported in less than 3%) when using proper kidney protection protocols with administration of positively charged amino acids infusion. The renal risk is lower for patients treated with Lutetium than those treated with Yttrium. Risk factors for severe renal deterioration are uncontrolled diabetes mellitus and hypertension [34, 36, 37].

Myelodysplastic syndrome (MDS) and leukemia can occur but are rare (about 1–3% of patients). Risk factors for hematologic side effects are age above 70, preexisting cytopenia, prior radiotherapy and treatment with alkylating agents [34, 36–38].

In the case of eventual future disease progression, one can consider repeating PRRT treatment as salvage therapy, although the response is less prominent than primary therapy [35, 39].

### Chemotherapy

In well-differentiated G1 & G2 NETs, several protocols of chemotherapy may be used, mainly containing alkylating agents (streptozotocin, dacarbazine and temozolomide), alone or in combination with an antimetabolite (5-fluorouracil, capecitabine). To date, there are no large phase III clinical trials providing solid evidence based recommendations for using the best regimen in the right sequence.

- ***Streptozocin-based chemotherapy***, in combination with 5-FU or doxorubicin, is an established therapeutic option in patients with PNETs, and is especially used in G2 progressive and/or associated with higher tumor burden.

For example, in a group of 96 patients with pNETs, mostly G2, the regimen of STZ and 5FU showed an objective response rate of 42.7%, stable disease in 40.6%, and progressive disease in 16.7%. The median time to progression was 19.4 months, and overall survival was 54.8 months [40].

The Uppsala group has recently published their experience with this regimen, in a retrospective evaluation of 133 medical records from the past 20–25 years, in patients with pNETs. The median survival was 51.9 months and the PFS was 23 months; complete response (CR), partial response (PR), stable disease (SD) and progressive disease (PD), were observed in 3, 25, 64 and 8%, respectively [41].

The toxicity profile of STZ + 5-FU is usually tolerable: adverse effects most frequently reported being nausea, fatigue, kidney toxicity, and less frequently bone marrow suppression [40].

b. ***Dacarbazine*** has been used alone or in combination with other agents to treat NETs. Most studies with dacarbazine showed response rates of 20–40%, PFS of 11–21 months, and median survival of 21–38 months. The most frequent toxicities were bone marrow suppression, nausea, vomiting [42].
c. ***Temozolomide (TMZ)***, a novel alkylating agent and oral derivative of dacarbazine, acts by methylation of the O6 position of guanine, resulting in DNA mismatch, and eventually apoptosis. It was suggested that low expression of the MGMT protein (involved in the DNA repair mechanism and associated with resistance to TMZ) may be a potential marker for predicting response to the TMZ; however, the role of MGMT is still controversial [43].

Temozolomide was evaluated as either monotherapy or in combination with other agents [43, 44]. The combination of capecitabine with temozolomide (CAPTEM) was postulated to have a synergistic effect for induction of apoptosis in NET cells. Two retrospective relatively small series of patients with well and moderately differentiated advanced PNETs treated with CAPTEM showed a radiologic response between 60 to 70% with a median PFS of 14 to 18 months [45, 46].

The use of TMZ alone or in combination (CAPTEM) for treating patients with advanced NETs has become common practice, and shows promising response rates with tolerable toxicities; however, most data comes from small retrospective or phase II prospective studies, with heterogeneous patients, regimens and doses. Pending data from a prospective trial of TMZ

Vs CAPTEM in progressive PNET (NCT01824875, www.clinicaltrials.gov) will help to address the remaining questions, regarding the best timing of treatment, best protocol, treatment duration, and the role of MGMT status evaluation as a biomarker, before and during the treatment.

### Immunotherapy

Immunotherapy is a rapidly evolving therapeutic field in different types of cancer. The immune checkpoint pathways acts physiologically to prevent activated T cells from an autoimmune activity. Programmed death receptor 1 (PD-1) is an inhibitory receptor on T cells that interacts with its’ ligands PD-L1 and PD-L2 to diminish the T cell antitumor response. In preliminary research, it was shown that antibodies against PD-1 or PD-L1 can enhance the T cell antitumor activity with acceptable safety and tolerability. Pembrolizumab is a potent, selective, humanized monoclonal antibody, with high affinity to PD-1 receptor [47]. While it was shown to be effective in other solid tumors (lung carcinoma, RCC, melanoma, Merkle cell carcinoma), there is very limited experience in patients with NETs, mainly suggesting stable disease as best response [48].

### Locoregional therapies

#### Thermal ablation

Thermal ablation is performed using radiofrequency ablation (RFA) or microwave ablation (MWA), delivering high frequency current to the lesions, inducing heat that destroys the proteins and leading to cellular death. The RFA is more frequently used than MWA, showing overall good clinical response, but as with surgery, the intrahepatic recurrence remains a problem.

The procedures can be performed percutaneously (under guidance of CT or US), or intraoperatively, usually combined with surgical resection. Classically, the indications for thermal ablation in liver metastases are less than 5 lesions, and less than 5 cm in size, however in NETs a more extensive metastatic spread is frequent, and the technique is used sometimes beyond these indications. Possible complications include abdominal pain, bile leakage, liver abscess, hemorrhage, bowel perforation [49].

#### Intra-arterial therapies (IAT)

Intra-arterial therapies are based on the knowledge that most liver metastases from NETs are hypervascular, and take their blood supply mainly from the hepatic artery, while the normal liver blood supply is from the portal vein [49].

- ***Trans-arterial embolization (TAE)*** causes ischemia and necrosis of the lesions, by injecting various particles (gelfoam, polyvinyl alcohol, microspheres).
- ***Trans-arterial chemoembolization (TACE)*** was developed in the 1990s, based on the rationale of embolizing the blood vessels just after delivering chemotherapy directly to the tumor cells by systemic injection. Both the high drug concentration, and ischemia of the cells, can enhance their response to the treatment. The most commonly used agents are doxorubicin and streptozotocin, alone or combined with other agents.

TAE and TACE were proven to reduce symptoms in 40–100% of NET patients in several series, with a morphological response in up to 94% of patients [50–54].

TACE-DEB (drug eluting beads) is another method, uses drug eluting beads that both embolize the arteries and slowly release the chemotherapy, usually doxorubicin.

The most common complications are fever, leukocytosis, abdominal pain, nausea, and elevated liver enzymes, known as the post embolization syndrome, which is usually transient. More severe complications are carcinoid crisis, liver failure, cholecystitis and liver abscess [49].

- ***Selective interval radiation therapy (SIRT)***- radioembolization using resin-based (Sirspheres) and glass-based (Theraspheres) micron sized particles, loaded with Yttrium-90 radioisotope, is increasingly being used, delivering high irradiation directly to the tumor. One series of 148 patients has shown positive response in 62.9% of patients, and stable disease in 22.7% [55].

High response rates, with improvement of both symptoms and overall survival, and tolerable toxicity were also shown in other series [56].

### External radiation

The improvement of radiation techniques allows delivering high dose of radiation locally to the lesions, with minimal damage to the surrounding tissues. However, as G1 and G2 NETs are usually considered radio-resistant, and the data about the activity and safety of radiation techniques in those tumors is limited, its use in NETs is limited mainly to palliation for bone or brain-metastases related symptoms or for pain control. [56–58].

### Systemic bone metastases directed therapies

The use of bisphosphonates in bone metastases of neuroendocrine tumors has been reported in several studies, however their effect on disease progression, pathologic fractures incidence, and pain control is yet to be evaluated [59–61].

Moreover, data on the use of denosumab, a monoclonal antibody with affinity for nuclear factor-kappa ligand (RANKL), in these patients is limited, and its possible beneficial effects are yet to be evaluated [59, 62].

### Liver transplantation

In the past, liver transplantation was used as salvage therapy, after failure of other treatments, with disappointing results. This option is reasonable with a curative purpose, as a well-planned procedure, with strict criteria of patient selection, when the expected 5-year survival rate is over 70%, and a 5-year disease free survival over 50% [63].

The Milan eligibility criteria for liver transplantation include age of 55 years and less, low grade GEP-NETs (Ki67 < 10%), primary tumor drained by the portal system (to be sure the primary site of metastatic spread was the liver), complete resection of primary tumor prior to transplantation, liver involvement of less than 50%, and stable disease or good response to previous treatment for 6 months before transplantation [64].

### Future directions

The discovery of the genome and DNA alterations led to major breakthroughs in medicine in general, and in cancer treatment specifically. However, epigenetic changes, which lead to heritable changes in gene expression, without modification of the DNA, including DNA methylation, histone modifications, and miRNAs, are also of great importance.

There is ongoing research for revealing epigenetic changes in NETs; several preliminary changes were found in NETs of the pancreas, lung and small intestine, with some of them suggesting poor prognosis, and justifying a more aggressive treatment approach. Furthermore, this approach may lead to the development of novel treatments, aimed at reversing the epigenetic modifications [65, 66].

## Conclusions

**Fig. 2:**
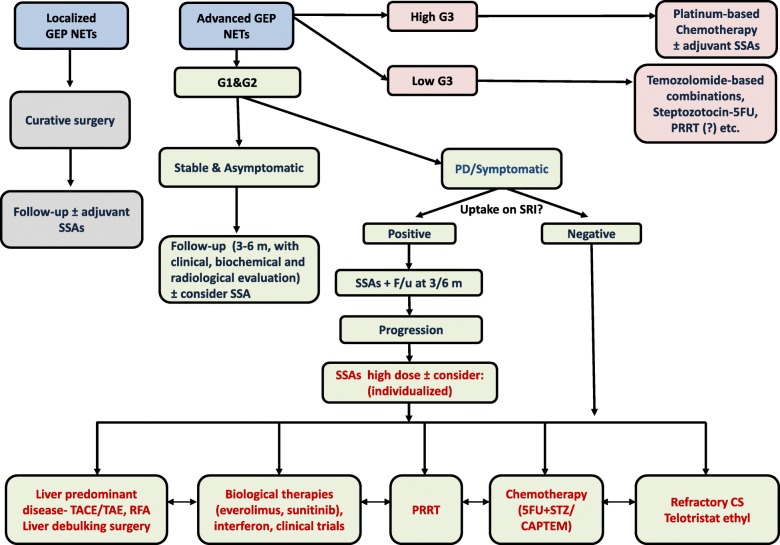
Possible algorithm for treatment approach in patients with GEP-NETs

In general, surgery is the first line treatment for localized neuroendocrine tumors, and it should be considered for palliation in metastatic or bulky disease. Evidence based treatments used recently for advanced G1 and G2 GEP NETs include somatostatin analogues (SSAs), the mTOR inhibitor everolimus, the tyrosine kinase inhibitor sunitinib, PRRT, and liver targeted therapies for localized unresectable disease (Table 1). For symptom control in advanced disease, SSAs are the mainstay of therapy, followed by the addition of telotristat ethyl in patients with uncontrolled carcinoid syndrome and other treatment modalities according to specified disease characteristics (Fig. 2) [67].

Since traditional treatments usually induce tumor stabilization for limited length of time, there is a great effort in developing novel approaches to overcome treatment-related resistance in patients with advanced and progressive NETs. The relatively small number of patients included in clinical studies, as well as the relatively slow course of the disease, make it difficult to evaluate the response rates for new therapies and their influence on survival. There are still many unmet needs in the therapeutic arsenal of NETs (e.g., the optimal sequencing of treatment modalities, exploration and validation of different biomarkers, etc). However, new insights into molecular alterations of neuroendocrine tumors should eventually improve the understanding of their complexity, facilitating a personalized approach and a successful treatment for each NET patient, in the near future.

## References

[1] Dasari A, Shen C, Halperin D, Zhao B, Zhou S, Xu Y, et al. Trends in the incidence, prevalence, and survival outcomes in patients with neuroendocrine tumors in the United States. JAMA Oncol. 2017; 10.1001/jamaoncol.2017.0589. 10.1001/jamaoncol.2017.0589 PMC5824320 28448665

[2] Grozinsky-Glasberg S, Grossman AB, Korbonits M (2008). The role of somatostatin analogues in the treatment of neuroendocrine tumours. Mol Cell Endocrinol.

[3] Raj N, Reidy-Lagunes D (2016). Systemic therapies for advanced pancreatic neuroendocrine tumors. Hematol Oncol Clin North Am.

[4] Kim JY, Hong SM (2016). Recent updates on neuroendocrine tumors from the gastrointestinal and Pancreatobiliary tracts. Arch Pathol Lab Med.

[5] Deppen SA, Blume J, Bobbey AJ, Shah C, Graham MM, Lee P, Delbeke D, Walker RC (2016). 68Ga-DOTATATE compared with 111In-DTPA-octreotide and conventional imaging for pulmonary and Gastroenteropancreatic neuroendocrine tumors: a systematic review and meta-analysis. J Nucl Med.

[6] Bousquet C, Lasfargues C, Chalabi M, Billah SM, Susini C, Vezzosi D (2012). Clinical review: current scientific rationale for the use of somatostatin analogs and mTOR inhibitors in neuroendocrine tumor therapy. J Clin Endocrinol Metab.

[7] Oberg KE, Reubi JC, Kwekkeboom DJ, Krenning EP (2010). Role of somatostatins in gastroenteropancreatic neuroendocrine tumor development and therapy. Gastroenterol.

[8] Alonso-Gordoa T, Capdevila J (2015). Grande E. GEP-NETs update: biotherapy for neuroendocrine tumours. Eur J Endocrinol.

[9] Chan JA, Kulke MH (2009). Progress in the treatment of neuroendocrine tumors. Curr Oncol Rep.

[10] Rinke A, Muller HH, Schade-Brittinger C, Klose KJ, Barth P, Wied M (2009). Placebo-controlled, double-blind, prospective, randomized study on the effect of octreotide LAR in the control of tumor growth in patients with metastatic neuroendocrine midgut tumors: a report from the PROMID study group. J Clin Oncol.

[11] Caplin ME, Pavel M, Cwikla JB, Phan AT, Raderer M, Sedlackova E (2014). Lanreotide in metastatic enteropancreatic neuroendocrine tumors. N Engl J Med.

[12] Pavel M, Valle JW, Eriksson B, Rinke A, Caplin M, Chen J (2017). ENETS consensus guidelines for the standards of Care in Neuroendocrine Neoplasms: systemic therapy - biotherapy and novel targeted agents. Neuroendocrinology.

[13] Wolin EM, Jarzab B, Eriksson B, Walter T, Toumpanakis C, Morse MA (2015). Phase III study of pasireotide long-acting release in patients with metastatic neuroendocrine tumors and carcinoid symptoms refractory to available somatostatin analogues. Drug Des, Dev Ther.

[14] Oberg K, Norheim I, Lind E, Alm G, Lundqvist G, Wide L (1986). Treatment of malignant carcinoid tumors with human leukocyte interferon: long-term results. Cancer Treat Rep.

[15] Oberg K (2012). Biotherapies for GEP-NETs. Best Pract Res Clin Gastroenterol.

[16] Pavel M, Horsch D, Caplin M, Ramage J, Seufferlein T, Valle J (2015). Telotristat etiprate for carcinoid syndrome: a single-arm, multicenter trial. J Clin Endocrinol Metab.

[17] Kulke MH, O'Dorisio T, Phan A, Bergsland E, Law L, Banks P (2014). Telotristat etiprate, a novel serotonin synthesis inhibitor, in patients with carcinoid syndrome and diarrhea not adequately controlled by octreotide. Endocr Relat Cancer.

[18] Kulke MH, Horsch D, Caplin ME, Anthony LB, Bergsland E, Oberg K (2017). Telotristat ethyl, a tryptophan hydroxylase inhibitor for the treatment of carcinoid syndrome. J Clin Oncol.

[19] Chan J, Kulke M (2014). Targeting the mTOR signaling pathway in neuroendocrine tumors. Curr Treat Options in Oncol.

[20] Svejda B, Kidd M, Kazberouk A, Lawrence B, Pfragner R, Modlin IM (2011). Limitations in small intestinal neuroendocrine tumor therapy by mTor kinase inhibition reflect growth factor-mediated PI3K feedback loop activation via ERK1/2 and AKT. Cancer.

[21] Yao JC, Phan AT, Chang DZ, Wolff RA, Hess K, Gupta S (2008). Efficacy of RAD001 (everolimus) and octreotide LAR in advanced low- to intermediate-grade neuroendocrine tumors: results of a phase II study. J Clin Oncol.

[22] Pavel ME, Hainsworth JD, Baudin E, Peeters M, Horsch D, Winkler RE (2011). Everolimus plus octreotide long-acting repeatable for the treatment of advanced neuroendocrine tumours associated with carcinoid syndrome (RADIANT-2): a randomised, placebo-controlled, phase 3 study. Lancet (London, England).

[23] Yao JC, Shah MH, Ito T, Bohas CL, Wolin EM, Van Cutsem E (2011). Everolimus for advanced pancreatic neuroendocrine tumors. N Engl J Med.

[24] Yao JC, Fazio N, Singh S, Buzzoni R, Carnaghi C, Wolin E (2016). Everolimus for the treatment of advanced, non-functional neuroendocrine tumours of the lung or gastrointestinal tract (RADIANT-4): a randomised, placebo-controlled, phase 3 study. Lancet (London, England)..

[25] Lombard-Bohas C, Yao JC, Hobday T, Van Cutsem E, Wolin EM, Panneerselvam A (2015). Impact of prior chemotherapy use on the efficacy of everolimus in patients with advanced pancreatic neuroendocrine tumors: a subgroup analysis of the phase III RADIANT-3 trial. Pancreas.

[26] Raymond E, Hobday T, Castellano D, Reidy-Lagunes D, Garcia-Carbonero R, Carrato A (2011). Therapy innovations: tyrosine kinase inhibitors for the treatment of pancreatic neuroendocrine tumors. Cancer Metastasis Rev.

[27] Capozzi M, Von Arx C, De Divitiis C, Ottaiano A, Tatangelo F, Romano GM (2016). Antiangiogenic therapy in pancreatic neuroendocrine tumors. Anticancer Res.

[28] Raymond E, Dahan L, Raoul JL, Bang YJ, Borbath I, Lombard-Bohas C (2011). Sunitinib malate for the treatment of pancreatic neuroendocrine tumors. N Engl J Med.

[29] Phan AT, Halperin DM, Chan JA, Fogelman DR, Hess KR, Malinowski P (2015). Pazopanib and depot octreotide in advanced, well-differentiated neuroendocrine tumours: a multicentre, single-group, phase 2 study. Lancet Oncol.

[30] Strosberg JR, Cives M, Hwang J, Weber T, Nickerson M, Atreya CE (2016). A phase II study of axitinib in advanced neuroendocrine tumors. Endocr Relat Cancer.

[31] Bergsma H, van Vliet EI, Teunissen JJ, Kam BL, de Herder WW, Peeters RP (2012). Peptide receptor radionuclide therapy (PRRT) for GEP-NETs. Best Pract Res Clin Gastroenterol.

[32] Brabander T, Teunissen JJ, Van Eijck CH, Franssen GJ, Feelders RA, de Herder WW (2016). Peptide receptor radionuclide therapy of neuroendocrine tumours. Best Pract Res Clin Endocrinol Metab.

[33] Strosberg J, El-Haddad G, Wolin E, Hendifar A, Yao J, Chasen B (2017). Phase 3 trial of 177Lu-Dotatate for midgut neuroendocrine tumors. N Engl J Med.

[34] Cives M, Strosberg J (2017). Radionuclide therapy for neuroendocrine tumors. Curr Oncol Rep.

[35] Kwekkeboom DJ, Krenning EP (2016). Peptide receptor radionuclide therapy in the treatment of neuroendocrine tumors. Hematol Oncol Clin North Am.

[36] Bodei L, Kidd M, Paganelli G, Grana CM, Drozdov I, Cremonesi M, Lepensky C, Kwekkeboom DJ, Baum RP, Krenning EP, Modlin IM (2015). Long-term tolerability of PRRT in 807 patients with neuroendocrine tumours: the value and limitations of clinical factors. Eur J Nucl Med Mol Imaging.

[37] Baum RP, Kulkarni HR, Singh A, Kaemmerer D, Mueller D, Prasad V, Hommann M, Robiller FC, Niepsch K, Franz H, Jochems A, Lambin P, Hörsch D (2018). Results and adverse events of personalized peptide receptor radionuclide therapy with 90Yttrium and 177Lutetium in 1048 patients with neuroendocrine neoplasms. Oncotarget.

[38] Kesavan M, Turner JH (2016). Myelotoxicity of peptide receptor radionuclide therapy of neuroendocrine tumors: a decade of experience. Cancer Biother Radiopharm.

[39] Vaughan E, Machta J, Walker M, Toumpanakis C, Caplin M, Navalkissoor S. Retreatment with peptide receptor radionuclide therapy in patients with progressing neuroendocrine tumours: efficacy and prognostic factors for response. Br J Radiol. 2018; 10.1259/bjr.20180041. 10.1259/bjr.20180041 PMC6475926 29513039

[40] Dilz LM, Denecke T, Steffen IG, Prasad V, von Weikersthal LF, Pape UF (2015). Streptozocin/5-fluorouracil chemotherapy is associated with durable response in patients with advanced pancreatic neuroendocrine tumours. Eur J Cancer (Oxford, England : 1990).

[41] Clewemar Antonodimitrakis P, Sundin A, Wassberg C, Granberg D, Skogseid B, Eriksson B (2016). Streptozocin and 5-fluorouracil for the treatment of pancreatic neuroendocrine tumors: efficacy, prognostic factors and toxicity. Neuroendocrinology.

[42] Okusaka T, Ueno H, Morizane C, Kondo S, Sakamoto Y, Takahashi H (2015). Cytotoxic chemotherapy for pancreatic neuroendocrine tumors. J Hepato-biliary-Pancreatic Sci.

[43] Koumarianou A, Kaltsas G, Kulke MH, Oberg K, Strosberg JR, Spada F (2015). Temozolomide in advanced neuroendocrine neoplasms: pharmacological and clinical aspects. Neuroendocrinology.

[44] Ekeblad S, Sundin A, Janson ET, Welin S, Granberg D, Kindmark H (2007). Temozolomide as monotherapy is effective in treatment of advanced malignant neuroendocrine tumors. Clin Cancer Res.

[45] Strosberg JR, Fine RL, Choi J, Nasir A, Coppola D, Chen DT (2011). First-line chemotherapy with capecitabine and temozolomide in patients with metastatic pancreatic endocrine carcinomas. Cancer.

[46] Fine RL, Gulati AP, Krantz BA, Moss RA, Schreibman S, Tsushima DA (2013). Capecitabine and temozolomide (CAPTEM) for metastatic, well-differentiated neuroendocrine cancers: the pancreas Center at Columbia University experience. Cancer Chemother Pharmacol.

[47] Patnaik A, Kang SP, Rasco D, Papadopoulos KP, Elassaiss-Schaap J, Beeram M (2015). Phase I study of Pembrolizumab (MK-3475; anti-PD-1 monoclonal antibody) in patients with advanced solid tumors. Clin Cancer Res.

[48] Pavel ME, Sers CWOMENINCANCERTHEMATICREVIEW (2016). Systemic therapies in neuroendocrine tumors and novel approaches toward personalized medicine. Endocr Relat Cancer.

[49] Zappa M, Abdel-Rehim M, Hentic O, Vullierme MP, Ruszniewski P, Vilgrain V (2012). Liver-directed therapies in liver metastases from neuroendocrine tumors of the gastrointestinal tract. Target Oncol.

[50] Kress O, Wagner HJ, Wied M, Klose KJ, Arnold R, Alfke H (2003). Transarterial chemoembolization of advanced liver metastases of neuroendocrine tumors--a retrospective single-center analysis. Digestion.

[51] Carrasco CH, Charnsangavej C, Ajani J, Samaan NA, Richli W, Wallace S (1986). The carcinoid syndrome: palliation by hepatic artery embolization. AJR Am J Roentgenol.

[52] Granberg D, Eriksson LG, Welin S, Kindmark H, Janson ET, Skogseid B (2007). Liver embolization with trisacryl gelatin microspheres (embosphere) in patients with neuroendocrine tumors. Acta Radiol (Stockholm, Sweden : 1987).

[53] Therasse E, Breittmayer F, Roche A, De Baere T, Indushekar S, Ducreux M (1993). Transcatheter chemoembolization of progressive carcinoid liver metastasis. Radiol.

[54] Grozinsky-Glasberg S, Kaltsas G, Kaltsatou M, Lev-Cohain N, Klimov A, Vergadis V, Uri I, Bloom AI, Gross DJ (2018). Hepatic intra-arterial therapies in metastatic neuroendocrine tumors: lessons from clinical practice. Endocrine.

[55] Kennedy AS, Dezarn WA, McNeillie P, Coldwell D, Nutting C, Carter D (2008). Radioembolization for unresectable neuroendocrine hepatic metastases using resin 90Y-microspheres: early results in 148 patients. Am J Clin Oncol.

[56] Mayo SC, Herman JM, Cosgrove D, Bhagat N, Kamel I, Geschwind JF (2013). Emerging approaches in the management of patients with neuroendocrine liver metastasis: role of liver-directed and systemic therapies. J Am Coll Surg.

[57] Chakravarthy A, Abrams RA (1995). Radiation therapy in the management of patients with malignant carcinoid tumors. Cancer.

[58] Chan DL, Thompson R, Lam M, Pavlakis N, Hallet J, Law C, Singh S, Myrehaug S (2018). External Beam Radiotherapy in the Treatment of Gastroenteropancreatic Neuroendocrine Tumours: A Systematic Review. Clin Oncol (R Coll Radiol).

[59] Van Loon K, Zhang L, Keiser J, Carrasco C, Glass K, Ramirez MT, Bobiak S, Nakakura EK, Venook AP, Shah MH, Bergsland EK (2015). Bone metastases and skeletal-related events from neuroendocrine tumors. Endocr Connect.

[60] Costa L, Major PP (2009). Effect of bisphosphonates on pain and quality of life in patients with bone metastases. Nat Clin Pract Oncol.

[61] Kos-Kudła B, O'Toole D, Falconi M, Gross D, Klöppel G, Sundin A, Ramage J, Oberg K, Wiedenmann B, Komminoth P, Van Custem E, Mallath M, Papotti M, Caplin M, Palma de Mallorca Consensus Conference Participants (2010). ENETS consensus guidelines for the management of bone and lung metastases from neuroendocrine tumors. Neuroendocrinology.

[62] Sohn W, Simiens MA, Jaeger K, Hutton S, Jang G (2014). The pharmacokinetics and pharmacodynamics of denosumab in patients with advanced solid tumours and bone metastases: a systematic review. Br J Clin Pharmacol.

[63] Grandhi MS, Lafaro KJ, Pawlik TM (2015). Role of Locoregional and systemic approaches for the treatment of patients with metastatic neuroendocrine tumors. J Gastrointest Surg.

[64] Mazzaferro V, Pulvirenti A, Coppa J (2007). Neuroendocrine tumors metastatic to the liver: how to select patients for liver transplantation?. J Hepatol.

[65] Stalberg P, Westin G, Thirlwell C (2016). Genetics and epigenetics in small intestinal neuroendocrine tumours. J Intern Med.

[66] Cives M, Simone V, Rizzo FM, Silvestris F (2016). NETs: organ-related epigenetic derangements and potential clinical applications. Oncotarget.

[67] Grozinskey-Glasberg G, Gross DJ (2012). New drugs in the therapy of neuroendocrine tumors. J Endocrinol Investig.

